# Dataset of a thermal model for the prediction of temperature fields during the creation of austenite/martensite mesostructured materials by localized laser treatments in a Fe-Ni-C alloy

**DOI:** 10.1016/j.dib.2023.109110

**Published:** 2023-04-03

**Authors:** H.J. Breukelman, M.J. Santofimia, J. Hidalgo

**Affiliations:** a Department of Materials Science and Engineering, Delft University of Technology, Mekelweg 2, 2628 CD Delft, the Netherlands; b Universidad de Castilla La Mancha, ETSII-INEI. DYPAM Research Group, Avda. Camilo José Cela s/n, Ciudad Real 13071, Spain

**Keywords:** Modelling temperature field, Steel, Local heat treatment, Flash heating

## Abstract

This work presents constitutive equations and a dataset of a thermal model for the prediction of temperature fields and heating rates during the application of localized laser treatments to a Fe-C-Ni alloy. The model considers transient material properties and the coupling between temperature and microstructure, with emphasis on the phase dependence of the thermal parameters and the hysteresis in the phase change. The model can predict temperature fields that are in agreement with the experimental microstructures at the laser-affected zones. This model can be applied to other materials exhibiting solid-state transformations upon the application of laser treatments.

**Specifications Table**

**Table:** 

Subject	Materials Science (General)
Specific subject area	Laser surface treatments
Type of data	Table, Figure, Equations
How data were acquired	Dilatometry experiment Microstructure characterization COMSOL Multiphysics software 5.3a JMATPro version 4.0
Data format	Raw analysed Filtered
Description of data collection	The input data was collected from both JMATPro and dilatometry as comma-separated value (csv) files, containing header information and records over the temperature range of interest. The other input for the model, a range of processing parameters for which it was validated, was based on an experimental full factorial design and is presented here in Table 1. The light microscope pictures after applying conditions in Table 1, are also included. Outputs from the COMSOL FEM were collected both as direct graphs and 3D figures from the software's graphical interface, as well as csv tables containing time/output values for various predefined sample points (probes) based on x,y, z-coordinates.
Data source location	The Delft University of Technology, Delft, The Netherlands
Data accessibility	The data will be published in: figshare Repository name: Dataset of a thermal model for the prediction of temperature fields of laser treatments in a Fe-Ni-C alloy Data identification number: 10.6084/m9.figshare.c.6278559 Direct URL to data: https://doi.org/10.6084/m9.figshare.c.6278559.v2
Related research article	H.J. Breukelman, M.J.M. Hermans, M.J. Santofimia, J. Hidalgo, Engineering austenite/martensite mesostructured materials by controlled localised laser treatments in a Fe-Ni-C alloy, Materials & Design Volume 227, 2023, 111,772. https://doi.org/10.1016/j.matdes.2023.111772

## Value of the Data

• The application of local solid-state laser heat treatment for the creation of patterned microstructures is a novel approach to both surface treatments and laser-based processing techniques in metals. The model presented here enables the assessment of the effect of process parameters on the highly local heat rate and peak temperature, directly related to the resulting microstructures.
• Material scientists and process technologists can utilize the presented data on new models and methods for developing novel heat treatments for patterned microstructures.
• This model is validated for the prediction of the extent of a laser-austenitized zone in the alloy under consideration and is assumed to be amenable to prediction within the same class of alloys when adjusted for the specific material parameters. Furthermore, the model has value in defining experimental campaigns for the design of patterned microstructures.
• The data presented here, as well as the recommendations in the related article, suggest a relevant further avenue of research by implementing coupling effects, notably those related to strain-induced phase transformations in future models.
• The performance of the model presented here is sufficient to resolve the heating rate gradients on short-distance scales of laser-affected microstructures. In the current model, it is expected that the implementation of a high number of coupling effects will demand higher-quality thermal data as the one provided here.
• The effects of solid-state transformations, exhibiting asymmetry between heating and cooling (phase change hysteresis) should be regarded as integral to the thermal property data (change in latent heat, enthalpy, density and thermal conductivity).

## 1 Objective

The main objective of this dataset is to provide additional results and data from that already supplied in the related published research article. In the related article, a thermal model was used to predict heating rates and temperature fields after the application of laser treatments to a Fe-Ni-C steel, in which laser process parameters were varied. This article collects the necessary material properties and parameters to feed the model, which was not fully described and provided in the related article. Besides, the related article only mentioned and discussed a representative number of laser conditions, 4 of the 20 conditions that were tested to validate the model. Here, the results in terms of microstructure characterization of the 20 evaluated conditions are also presented, which complements and supports the discussion and conclusions extracted in the related article.

## 2 Data Description

This section describes the relevant data input of a thermal model implemented in COMSOL Multiphysics software 5.3a for the prediction of temperature fields and heating rates during the application of localized laser treatments to a Fe-C-Ni alloy. The input for the model consists of laser processing parameters and material thermal properties that are collected in the raw data files. Besides, the raw data contains resulting microstructures after the application of laser parameters that are used for the validation of the aforementioned thermal model.

### 2.1 Laser processing parameters

Table 1 shows the range of processing parameters for which the model was validated based on a sequential experimental factorial design optimization applied to 4 mm thickness specimens. A microstructural inspection was done after the application of different processing parameters which was contrasted with the temperature fields predicted by the model. The observation of three different microstructure regions, Z_A_, Z_B_ and Z_C_ allowed making correspondence with the temperatures achieved at different locations of the working piece. Z_A_ denotes a region in which melting was observed (temperature higher than 1710 K for the studied material, estimated by ThermoCalc^Ⓡ^), Z_B_ delimits a region in which martensite to austenite transformation occurred (temperature higher than 822 K, obtained by dilatometry experiments), and Z_C_ is a transition region to the base martensitic microstructure.

**Table 1:** Combination of processing parameters for which the model is validated. P is the laser power, v is the travel speed of the laser concerning the working piece, rspot is the spot size. ZA denotes a region in which melting was observed, ZB delimits a region in which martensite to austenite transformation occurred, and ZC is a transition region to the base martensitic microstructure.

LINE	*P* [W]	*V* [mm/s]	*r_spot_* [mm]	Z_A_ [μm]	Z_B_ [μm]	Z_C_ [μm]
1	50	25	0.45	0	252	79
2	50	42	0.45	0	168	76
3	50	58	0.45	0	0	180
4	50	75	0.45	0	0	140*
5	75	75	0.45	0⁎⁎	220	45
6	100	25	0.45	83	264	79
7	100	75	0.45	0	213	43
8	100	100	0.45	0	143	53
9	300	25	0.45	291	245	107
10	200	2.5	0.78	0	1578	397*
11	200	5	0.78	0	1158	612
12	200	25	0.78	0	0	994
13	200	75	0.78	0	0	606
14	400	5	0.78	706	1057	945
15	400	20	0.78	0	1113	225
16	400	25	0.78	0	957	353
17	400	75	0.78	0	0	553*
18	200	5	1.02	0	0	2060
19	400	5	1.02	0	1924	944*
20	400	25	1.02	0	0	1706

⁎ inhomogeneous.

⁎⁎ inconsistent melting.

**Fig. 1:**
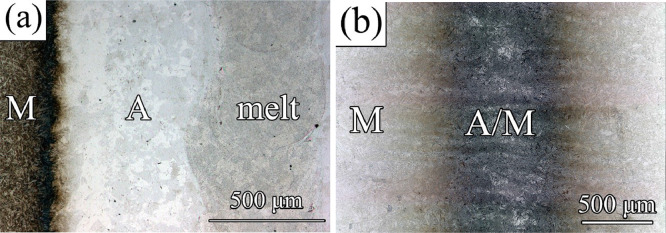
Microstructures of conditions (a) LINE 9 and (b) LINE 17 in Table 1. M = martensite, A = austenite.

Some examples of microstructures formed upon the application of laser treatment conditions in Table 1 can be seen in Fig. 1. The micrographs for the rest of the conditions can be consulted in the repository associated with this publication.

### 2.2 Material transient properties

The data used to consider the temperature dependence of density, heat capacity and thermal conductivity in the thermal model is described here. The density and the heat capacity are treated as phase-dependent parameters by the implementation of phase parameter Θ in Eq. (1.a) and Eq. (2). Θ takes the value 1 and 0 indicating martensite or austenite phases respectively as detailed explained in Section 2.

While the temperature-dependent behaviour of the thermal conductivity was implemented in the model, no satisfactory data could be obtained to describe the phase dependence of the thermal conductivity. Therefore, the implementation of the phase parameter for thermal conductivity was considered out of scope for this edition of the model.

**Fig. 2:**
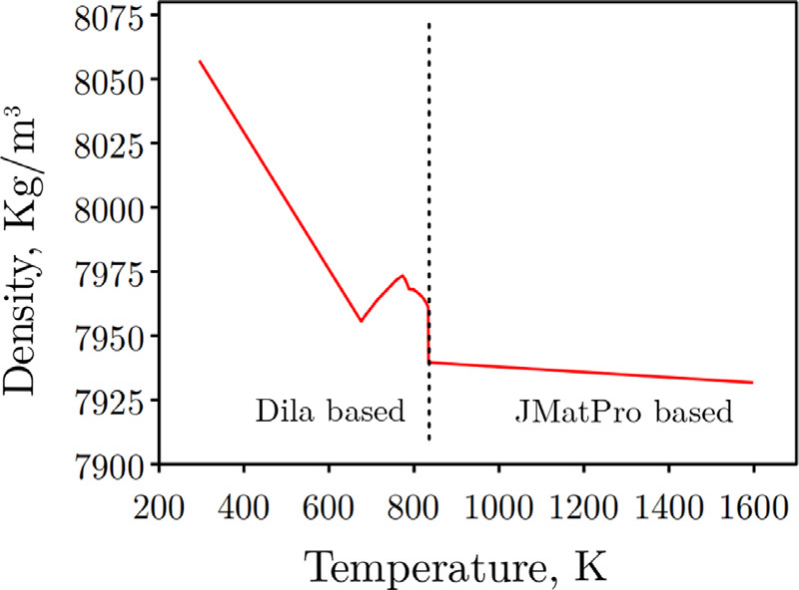
Variation of density with temperature.

Fig. 2 shows the variation of density (*ρ*) with temperature used to consider the transient variation of this property during simulations. This data is based on the results from dilatometry and data obtained from JMatPro and was fitted to different polynomial type Eqs. (1.b to g) depending on the temperature range:

(1.a) $$\rho =\Theta \rho_{mar}+\left(1-\Theta \right)\rho_{aus}$$

(1.b) $$\rho_{mar}=8058\;if\;T\;\epsilon \left(270,293\right)K$$

(1.c) $$\rho_{mar}=\frac{8058}{1+33.75*10^{-6}*\left(T-293\right)}\;if\;T\;\epsilon \;\left(293,673\right)K$$

(1.d) $$\begin{array}{cc} \rho_{mar} & =\frac{7955}{1+3*\left(-0.0000210^{-6}*{\left(T-673\right)}^{2}+0.0310^{-6}*\left(T-673\right)-10.210^{-6}\right)*\left(T-673\right)}\\ & if\;T\;\epsilon \;\left(673,773\right)K \end{array}$$

(1.e) $$\begin{array}{cc} \rho_{mar} & =\frac{7973}{1+3*\left(0.000356*10^{-6}*{\left(T-773\right)}^{2}+0.813510^{-6}*\left(T-673\right)-4.110^{-6}\right)*\left(T-773\right)}\\ & if\;T\epsilon \left(773,787\right)K \end{array}$$

(1.f) $$\begin{array}{cc} \rho_{mar} & =\frac{7968}{1+3*\left(0.00010310^{-6}*{\left(T-673\right)}^{2}+0.17310^{-6}*\left(T-673\right)-4.110^{-6}\right)*\left(T-787\right)}\\ & if\;T\;\epsilon \;\left(787,833\right)K \end{array}$$

(1.g) $$\rho_{aus}=\frac{7939}{1+3*\left(0,4392*10^{-6}*\left(T-833\right)\right)}\;if\;T\;\epsilon \;\left(833,1600\right)K$$

where *ρ_mar_* is the density of the martensite phase, *ρ_aus_* is the density of the austenite.

**Fig. 3:**
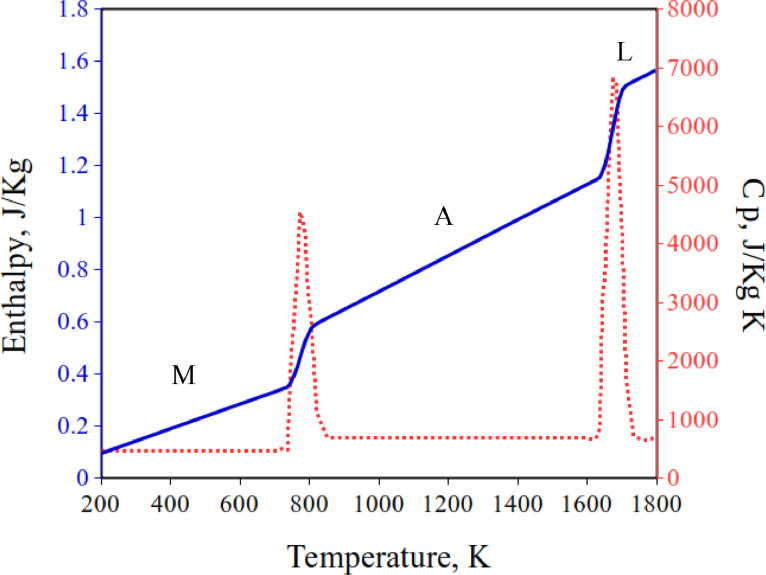
Variation of enthalpy and heat capacity at constant pressure with temperature. M refers to martensite, A to austenite and L to liquid phases.

*Cp* is the derivative of the enthalpy *H* with respect to temperature at constant pressure, as expressed in Eq. (2). This equation is valid at temperatures below the formation of the liquid phase. An analogous equation can be formulated for the transition of austenite to the liquid phase. Hence, *Cp* was simplified to only the major phase transitions and 3 constants for the slope of the enthalpy of each phase (Δ*H_martensite_* → Δ*H_austenite_* → Δ*H_liquid_*) and its variation with temperature is shown in Fig. 3. The enthalpy of each phase is assumed to linearly vary with temperature and is extracted from JMatPro.

(2) $$c_{p}=\frac{\delta }{\delta T}\left(\Theta *\Delta H_{mar}+\left(1-\Theta \right)\Delta H_{aus}\right)$$

**Fig. 4:**
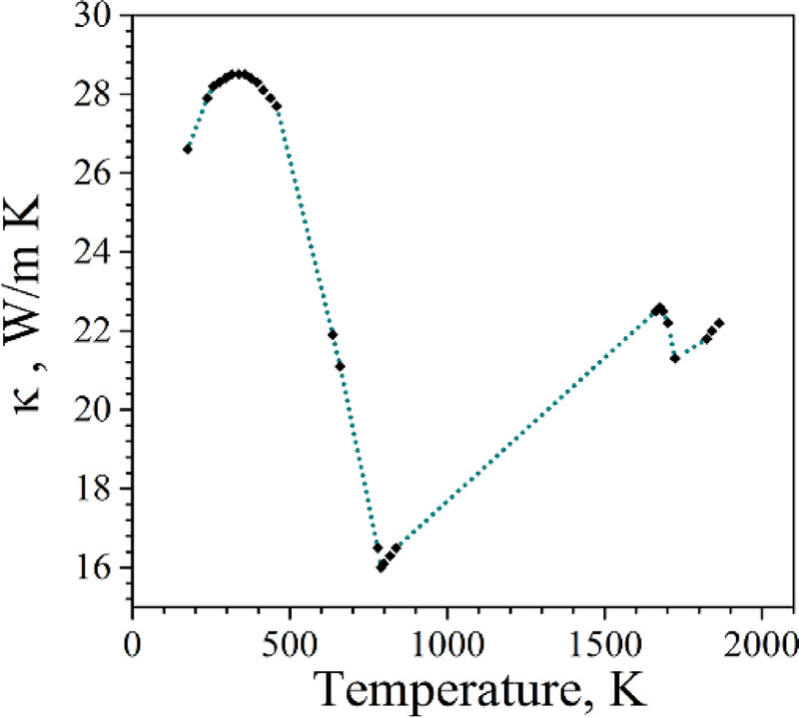
Variation of heat conductivity with temperature.

Finally, variation of κ with temperature was obtained by linear interpolation of data points generated by JmatPro, as shown in Fig. 4.

## 3 Experimental Design, Materials and Methods

This section describes the methods used to obtain relevant input for the thermal model, also described in this section, and its implementation in COMSOL Multiphysics software 5.3a. The resulting COMSOL file, available at the figshare repository, contains all the relevant information necessary for conducting laser treatment simulations in a working piece of dimensions 4 mm thick, 40 mm length and 12 mm wide made of a model metastable austenitic alloy of composition in wt.% 74.78Fe-0.2C-25Ni-0.02Mn.

**Fig. 5:**
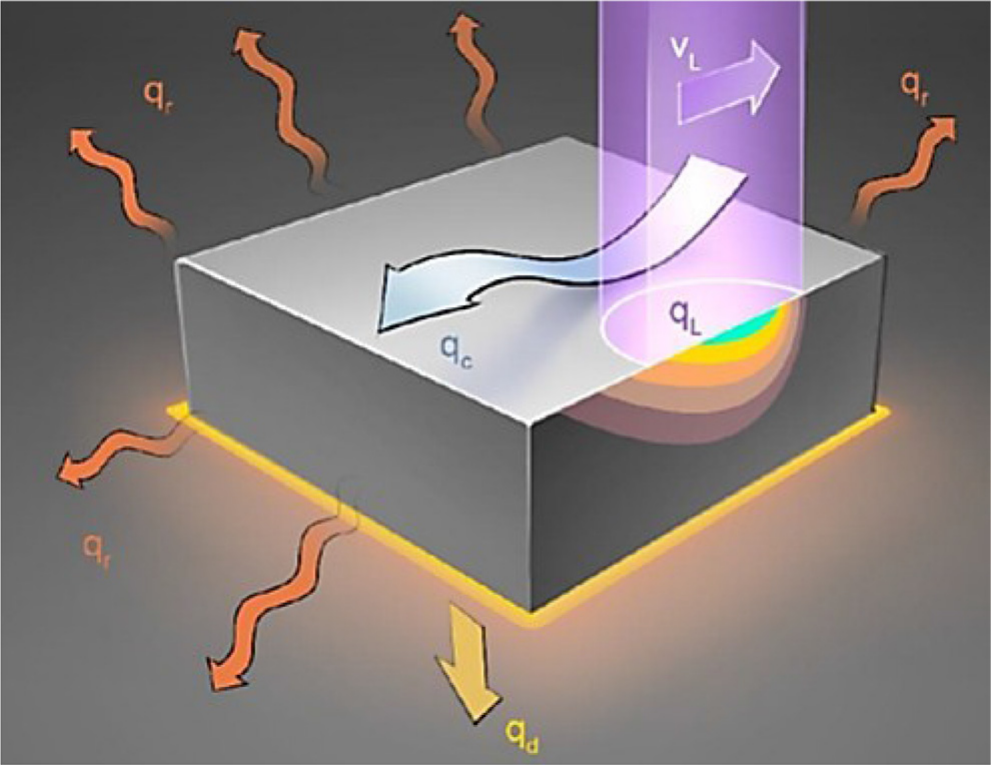
Schematic of the different heat fluxes in and out of the working piece.

The thermal model is based on the following heat equation describing the temperature (*T*, in K) change in time (*t*, in s) at a general point in the domain described by the coordinates *x, y, z*:

(3) $$\frac{\partial T}{\partial t}=\frac{\kappa }{\rho C_{p}}\left(\frac{\partial^{2}T}{\partial x^{2}}+\frac{\partial^{2}T}{\partial y^{2}}+\frac{\partial^{2}T}{\partial z^{2}}\right)+\frac{q}{\rho C_{p}}$$

where *κ*, the heat conductivity in W/m K, *C_p_*, the specific heat capacity at constant pressure with units J/kg K, and *ρ,* the density in kg/m^3^, are intrinsic material properties that depend on temperature. The variable *q* denotes the overall heat flux, which combines the different heat fluxes in and out of the working piece considered in the modelling of the laser treatments as schematized in Fig. 5. It includes the laser heat flux (*q_L_*), the radiative heat loss to the environment (*q_r_*), and the heat loss due to natural convection (*q_c_*) and thermal contact with a resting block (*q_d_*). In the numerical model, these fluxes are defined for specific boundaries, using the appropriate equations.

The boundary condition of particular interest to this work is *q_L_*, describing the power density of the laser beam directly in terms of the laser processing parameters, laser power, *P* in Watts and the laser spot radius, *r*_spot_, in meters. *q_L_* is modelled adequately using Gaussian distributions [1], the simplest of which can be described with Eq. 4:

(4) $$q_{L}\left(\left[\frac{W}{m^{2}}\right]\right)=\frac{A*8P}{4\pi r_{spot}^{2}}exp\left(-{\left(\frac{\sqrt{2}}{r_{spot}}\right)}^{2}\left(x{\left(v\right)}^{2}+y^{2}\right)\right)$$

Also incorporated is the location of the laser spot along the x-axis, which is a function of laser speed *v* [m/s]. The presence of *x* and *y* coordinates in the negative exponent results in a gradual decay of the intensity with increasing distance from the centre of the laser focal spot located at (*x(v), y*). The interaction of the laser radiation with the metallic surface is modulated by the absorptivity, *A*, which can be expressed as the ratio of absorbed power over laser power [2]. An absorptivity of 0.8 was selected for the application of the model on a Fe-Ni-C alloy with significant surface roughness based on the range of values reported by Bergstrom for a variety of steels and surface conditions [2]. Absorptivity was maintained constant with temperature in the present model.

(5) $$q_{r}=\varepsilon \sigma \left(T_{ambient}^{4}-T^{4}\right)$$

The radiative heat flux in Eq. 5 [3], describes the net blackbody radiation from the workpiece (at temperature, *T*) to the environment (at temperature, *T*_ambient_) as a function of the black body emissivity of the material, ε, and the Stefan-Boltzmann constant σ. The emissivity can be considered equal to the absorptivity in thermal equilibrium. Thermal equilibrium condition holds in most of the working piece, except in the region directly affected by the laser. Nevertheless, emissivity was taken to be equal to the absorptivity for all modelled conditions, which is a reasonable assumption as radiative outward heat flux can be considered negligible relative to the total heat input and the self-quenching internal conduction.

(6) $$q_{c}=h_{c}\left(T_{ambient}-T\right)$$

(7) $$-n_{d}\cdot q_{d}=-h\left(T_{ambient}-T\right)+rQ_{b}$$

(8) $$h_{c}=\frac{k}{l}0.15Ra_{L}^{1/3}$$

(9) $$h=h_{g}+h_{cont}$$

Natural convection (Eq. 6) and thermal contact (Eq. 7) are both based on Newton's Law of Cooling and depend on the temperature difference between the workpiece (*T*) and environment (*T_ambient_*), and the heat transfer coefficient, *h*. For the convection case (Eq. 8), *h_c_* is based on the Raleigh number, *Ra_L_*, which describes the ratio between conductive and convective forces in the fluid in contact with the workpiece. In the case of thermal contact heat flux (Eq. 9), *h* is made up of a contribution *h_cont_* representing the direct contact between the workpiece and the resting block, and *h_g_* representing the gaps caused by the surface roughness of the resting block and the workpiece. *n_d_* is the normal vector to the upper surface, describing the direction of the heat flux rigorously, even when the surface roughness is taken into account. *r* is the friction heat partition coefficient, and *Q_b_* is the generated heat of friction generated from two adjoining surfaces expanding asynchronously due to a temperature and thermal expansion coefficient difference. The friction term in (Eq. 7) can be neglected in the present case.

The model was tested experimentally by applying the laser conditions detailed in Table 1. Hence, the material properties implemented in the model are considered according to the initial and final microstructures achieved after the application of laser treatments. By cryogenic quenching, a base martensitic microstructure is formed which is the initial condition in the experiments and for data acquisition in the model. The data range presented here covers the heating of the base martensitic material by a laser source to above-ambient temperatures up to 1370 K, followed by cooling to room temperature. These temperatures were also contrasted experimentally.

For the application of the model, firstly, the geometry of the working piece and a resting block along with the boundary conditions are defined, which can be altered for specific applications. The COMSOL's material property library was used to obtain reasonably accurate model input in several non-critical aspects, mostly boundary conditions not directly related to the interaction of the workpiece with the laser and the workpiece's thermal properties. This decision is justified based on the highly local heat flux and the relatively low heat input compared to the total workpiece volume. Some of the considerations are described next.

A standard copper black with dimensions significantly wider, thicker, and longer than the workpiece was selected for the model component that represents the working surface on which samples were placed for heat treatment. To represent the boundary between the workpiece and the copper working surface, a thermal contact boundary was modelled based on the Thermal Contact node available in the Heat Transfer Module of COMSOL. The solid-solid conduction component was modelled using the Cooper – Mikic – Yovanovich correlation based on the renowned work of these authors [4]. The surface roughness parameter was 1 µm, and its slope 0.4. The gap gas conductance of the thermal contact boundary was based on the parallel plate gap gas conductance approach, with thermal parameters based on air at standard temperature and pressure. Radiative heat transport was neglected at the thermal contact boundary.

A heat flux node was implemented for external natural convective cooling of the upside of a horizontal plate. The characteristics length scale was determined based on the geometry of the workpiece (area divided by perimeter). The parameters of the cooling fluid were those of air, with standard temperature and pressure conditions at an infinite distance from the workpiece.

The laser process parameters are *P, v* and *r_spot_* of the laser. They can be varied to tailor the heat supplied by the laser and thus the transient temperature fields and heating rates along the working specimen. This is crucial to have control over the microstructures engineered in patterns as the main application of this model. Input from the laser processing parameters is based on a factorial experimental design of Table 1 for which melting was not observed in the model alloy after grinding up to a P80 grid.

Absorptivity can be adjusted to different materials and surface conditions in the present model. The absorbed power is not easily measured, nor is there agreement on the validity of theoretical models for the calculation of absorptivity [5]. A crucial step in the modelling approach is accounting for the loss in accuracy caused by misestimation of the absorptivity because a slight change in absorptivity has a strong effect on the simulated results. Besides the modification of laser processing parameters, the high sensitivity of the laser treatment to changes in absorptivity enables to play with this parameter to tailor the extent of the temperature field. In this regard, the model described in this publication also offers the opportunity to assess the adequate material absorptivity, which can be later adjusted by modifying the specimen surface finish or applying absorptivity-altering coatings.

The model also considers transient material properties, heat conductivity (*κ*), specific heat capacity at constant pressure (*C_p_*), and density (*ρ*). The variation of these material properties with temperature is accounted for based on the data obtained from JMatPro 4.0, and adjusted based on the results from dilatometry w.r.t. the phase transformation temperatures following the procedure described in the accompanying paper and using a Bähr DIL 805 A/D/T dilatometer. These properties govern the heat conduction behaviour in solid materials and are themselves temperature and phase-dependent [6,7]. In this work, these issues have been addressed by:

• Implementing a density-temperature curve based on dilatometer measurements and JMatPro for temperatures higher than 1373 K.
• The use of Physical-Model assisted software, JMatPro, for *Cp* and *κ.*
• Adjustments to JMatPro data on *Cp* based on the phase transformation temperatures observed from dilatometry.

The material thermal properties, for which values were obtained over the full temperature range at 20 K intervals, have been sampled to contain a limited number of data points. This approach was taken to limit computational time, due to the effect that local discontinuities have on FEM computational requirements. This down-scoping was limited to linear regions in the data-temperature curves, with higher data resolution preserved in the non-linear zones to retain accuracy.

Furthermore, a phase change hysteresis should be reflected in the model, where the nodes that have experienced transformation to austenite by heating to temperatures higher than the austenite finish temperature, *A_f_*, should be assigned the thermal properties appropriate to that phase. To this end, a phase parameter Θ ∈ [0,1] was defined, where 1 denotes the initial martensitic microstructure, 0 a fully austenitic microstructure, and any ratio a mixture of these two. The nodal property Θ was assigned based on conditional statements, relating the phase nature to the highest attained temperature at that node. This ensures that once a temperature above *A_f_* is reached in a node, the part of the model it represents will be treated as austenite, even when the temperature falls below the austenite start temperature, *A_s_*_,_ subsequently. The thermophysical properties of either phase were assigned to a node based on this phase parameter.

The main output from the model consists of spatially continuous Time-Temperature data, from which local peak temperatures, local heating rates, and local cooling rates, can be obtained and compared to experimental data. Furthermore, based on the phase parameter Θ, an assessment can be made of the extent of the laser-austenitized zone. For the microstructure inspection, specimens subjected to laser conditions in Table 1 were grounded and polished up to 1 μm diamond paste and etched with Kalling's #2 reagent (5 gr CuCl_2_ + 100 ml of HCl at 33% + 100 ml Ethyl alcohol). Light optical microscopy (LOM) was performed in a Keyence VHX-100 digital microscope, making a composite image of several high-resolution images covering all the laser-affected regions of the specimens. Based on Table 1 results and for the range of laser processing parameters and temperatures described here, it can be concluded that the model can predict temperature fields that are in agreement with the experimental microstructures at the laser-affected zones, which is thoroughly discussed in the journal article accompanying this data set.

## Ethics Statements

This work does not involve studies with animals and humans and complies with the ethical requirements for publication in Data in Brief.

## CRediT authorship contribution statement

**H.J. Breukelman:** Writing – original draft, Methodology, Software. **M.J. Santofimia:** Supervision, Writing – review & editing. **J. Hidalgo:** Conceptualization, Validation, Writing – original draft, Visualization, Supervision.

## Declaration of Competing Interest

The authors declare that they have no known competing financial interests or personal relationships that could have appeared to influence the work reported in this paper.

## Data Availability

Dataset of a thermal model for the prediction of temperature fields of laser treatments in a Fe-Ni-C alloy (Original data) (figshare).
